# Development of sarcosine quantification in urine based on enzyme-coupled colorimetric method for prostate cancer diagnosis

**DOI:** 10.17179/excli2018-1245

**Published:** 2018-05-17

**Authors:** Vichanan Yamkamon, Benjarong Phakdee, Sakda Yainoy, Thummaruk Suksrichawalit, Tararat Tatanandana, Premsant Sangkum, Warawan Eiamphungporn

**Affiliations:** 1 Department of Clinical Microscopy, Faculty of Medical Technology, Mahidol University, Bangkok 10700, Thailand; 2 Department of Clinical Microbiology and Applied Technology, Faculty of Medical Technology, Mahidol University, Bangkok 10700, Thailand; 3 Center of Data Mining and Biomedical Informatics, Faculty of Medical Technology, Mahidol University, Bangkok 10700, Thailand; 4 Department of Clinical Chemistry, Faculty of Medical Technology, Mahidol University, Bangkok 10700, Thailand; 5 Department of Surgery, Faculty of Medicine Ramathibodi Hospital, Mahidol University, Bangkok 10400, Thailand

**Keywords:** colorimetric assay, sarcosine, prostate cancer, sarcosine oxidase, urine

## Abstract

An enzyme-coupled colorimetric assay for quantification of urinary sarcosine was developed. The proposed method is a specific reaction based on hydrogen peroxide (H_2_O_2_) formation via sarcosine oxidase (SOX). The liberated H_2_O_2 _reacts with Amplex Red in the presence of horseradish peroxidase (HRP) to produce the red-fluorescent oxidation product, resorufin, which can be measured spectrophotometrically (OD570). The method was performed in the 96-well microtiter plate. Reaction conditions, such as pH and reaction time were optimized. At the optimum conditions, the limit of detection (LOD) and quantification (LOQ) were found to be 0.7 and 1 µM, respectively. A good linearity was revealed with a coefficient of 0.990. The assay showed no significant interference from ascorbic acid, glucose and bilirubin. In addition, it is extremely specific for sarcosine rather than other amino acids. The determination of sarcosine in human urine displayed high accuracy and good reproducibility. This method is promising to differentiate prostate cancer patients from healthy subjects according to urinary sarcosine level. Altogether, this study provides a rapid, simple and specific tool to determine urinary sarcosine which could be useful for prostate cancer diagnosis.

## Introduction

Prostate cancer (PCa) is the second most diagnosed cancer in men, particularly those over 50 years of age. Although prostate cancer can be slow growing, the disease accounts for the leading cause of cancer-related deaths in men worldwide. Currently, the digital rectal examination (DRE) and the prostate specific antigen (PSA) blood test are the two screening methods of PCa recommended by the American Cancer Society (Smith et al., 2004[30]). However, the above methods have the limitations regarding their sensitivity, specificity and reliability (Ankerst and Thompson, 2006[1]; Roobol et al., 2007[27]; Aslan et al., 2011[3]). As a result, using these methods can cause misdiagnosis, leading to delayed treatment or overtreatment (Loeb and Catalona, 2010[22]). Consequently, many efforts have been conducted to discover a potential biomarker for a more accurate diagnosis and for both fine selections of the therapeutic regimens and timely monitoring of the response to therapy (Jamaspishvili et al., 2010[16]; Clint Cary and Cooperberg, 2013[12]). Recently, many studies demonstrated an important role of sarcosine (N-methylglycine), an intermediate product in the synthesis and degradation of glycine, in PCa progression (Sreekumar et al., 2009[31]; Khan et al., 2013[19]). Sarcosine has been proposed as a PCa marker candidate since it is highly elevated during PCa progression to metastasis, whereas it was not detected or presented at very low concentrations in the urine of healthy individuals (Sreekumar et al., 2009[31]; Bianchi et al., 2011[4]; Khan et al., 2013[19]). Therefore, effective diagnostic tools for the detection of sarcosine in urine, a noninvasive specimen, are highly desirable. So far, urinary sarcosine determination has been achieved using different approaches including LC-MS (Jiang et al., 2010[17]), GC-MS (Cavaliere et al., 2011[9]; Shamsipur et al., 2013[29]), fluorometric method (Burton et al., 2012[6]), supramolecular sensor based on a functionalized silicon surface (Biavardi et al., 2012[5]), and reflectometric nanosensor (Diltemiz and Uslu, 2015[13]). However, these techniques possess practical disadvantages, such as time-consuming, complex sample preparation, skilled operator requirements and high cost, which are not appropriate for routine analysis. Moreover, some of the above mentioned methods showed interference by the presence of other amino acids, such as alanine and glycine in urine or due to unspecific reactions with other urinary analytes (Schalken, 2010[28]; Meyer et al., 2011[24]). Therefore, the selective and sensitive methods are needed for sarcosine detection. To overcome these drawbacks, colorimetric methods based on substrate-specific enzymes have been proposed considering their cheapness, simplicity, high sensitivity and selectivity. Sarcosine oxidase (SOX) is an enzyme that catalyzes the oxidative demethylation of sarcosine to glycine, formaldehyde and hydrogen peroxide (H_2_O_2_). Although direct determination of sarcosine can be difficult, indirect quantification can be accomplished by this enzymatic reaction (Burton et al., 2012[6]). However, some colorimetric assays have a problem when the complex matrices, urine and blood, were applied for analysis, such as the assay using the sequential reaction of SOX and peroxidase with 4-aminoantipyrine as a chromogen (Kinoshita and Hiraga, 1980[20]). Using SOX for sarcosine detection in biological samples, such as electrochemical biosensors and nanoparticle-based assay has been previously described in the literature (Yadav et al., 2012[37]; Lan et al., 2014[21]; Rebelo et al., 2014[26]). These assays might have a disadvantage due to lot-to-lot variation of synthesis resulting in the non-reproducibility of the test. Moreover, in case of nanoparticle-based assay, the dispersion degree of nanoparticles directly affects the catalytic stability and the assay is still prolonged to perform.

**Figure 1:**
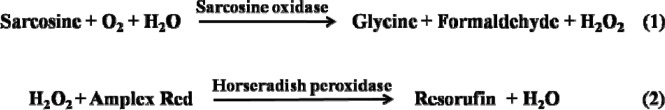
Colorimetric assay for quantification of sarcosine used in this study

In this study, a simple, rapid and sensitive colorimetric assay based on the catalytic reaction of SOX and horseradish peroxidase (HRP) was developed and evaluated for sarcosine determination in real urine samples. Our assay was performed in a 96-well microtiter plate and measured the absorbance using UV-visible spectrophotometer which is widely used in routine analysis. Amplex Red was used as a substrate in the assay according to its advantages of being colorless in aqueous solution, low background, great stability, high sensitivity and strong anti-interference ability (Zhou et al., 1997[39]; Votyakova and Reynolds, 2004[34]). For determination of sarcosine, the enzymatic reaction is shown in Figure 1(Fig. 1). The optimized procedure was then applied to examine the sarcosine in real samples from healthy volunteers and patients diagnosed with prostate cancer.

## Materials and Methods

### Chemicals and reagents

All chemicals were of analytical grade. Ampicillin, isopropyl β-D-1-thiogalactopyranoside (IPTG) and imidazole were obtained from Bio Basic Inc. (Markhem, ON, Canada). Sarcosine, Amplex Red (10-acetyl-3,7-dihydroxyphenoxazine) and HRP were ordered from Sigma-Aldrich (Saint Louis, MO, ^U^SA). H_2_O_2_^a^nd phenol were from MERCK (Darmstadt, Germany). Milli-Q system ultra-pure water was used in all experiments. Pfu DNA polymerase was obtained from Promega (Madison, WI, USA). Restriction endonucleases and T4 ligase were purchased from New England Biolabs, Inc. (Ipswich, MA, USA).

### Gene, plasmid and bacterial strains

The coding sequence of SOX from *Bacillus *BSD-8 was synthesized by Integrated DNA Technologies, Inc. (Coralville, IA, USA). pET-20b(+) expression vector, *E. coli *strain NovaBlue and BL21(DE3) were obtained from Novagen (Darmstadt, Germany).

### Production of recombinant sarcosine oxidase (SOX)

The open reading frame of *sox* gene was amplified by PCR with the forward primer (5'-ACCATATGAGCACGCATTTTGATG-3', underlined is NdeI restriction site) and the reverse primer (5'-CAACTCGAGTTTTGC- TGCTTCCTT-3', underlined is XhoI restriction site). The 1,164 bp PCR product was cut with NdeI and XhoI and ligated into the pET-20b(+) vector pretreated with the same restriction enzymes. The recombinant plasmid was verified by DNA sequencing. The protein expression was carried out in *E. coli* BL21(DE3). The recombinant strain was grown overnight at 37 °C in LB medium containing 100 μg/ml ampicillin. The culture was transferred into Terrific Broth (TB) with the same antibiotic concentration and incubated at 37 °C, 150 rpm until OD600 reached 0.5. The SOX expression was induced with 1 mM of IPTG for an additional 16 h at 37 °C. Cells were harvested by centrifugation and suspended in buffer A (50 mM phosphate buffer, pH 7.4) followed by sonication. The lysates were cleared by centrifugation. The supernatant was filtered and loaded on a Ni-NTA agarose column which pre-equilibrated with buffer A (50 mM phosphate buffer, pH 7.4) with ÄKTA prime protein purification system (GE healthcare life sciences, UK). After elution with a linear gradient of buffer B (buffer A + 1 M imidazole), the target protein containing fractions were combined. The imidazole was removed and purified protein was concentrated by an Amicon Ultra 10,000-MWCO filter (EMD Millipore Corp., MA, USA). Protein molecular weight and purity under denaturing condition were determined by SDS-PAGE. Protein concentration was measured by Bradford method, before storage at -80 °C.

### Optimization of two-enzyme coupled assay for sarcosine measurement

Standard solutions of sarcosine were prepared at concentration ranging from 1-200 µM (1, 2, 3, 4, 5, 10, 20, 30, 40, 50, 100 and 200 µM) using distilled water. The reactions were performed in 96-well microtiter plates (SPL Life Sciences Co., Ltd., Gyeonggi-do, South Korea) and the absorbance was measured by a microplate reader (Synergy HTX multi-mode, BioTek, Winooski, VT, USA). The total volume of each reaction was 200 µL. Reactions were performed by adding 127.5 µL of 50 mM sodium phosphate buffer (pH 7.5), 20 µL of sarcosine solution, 12.5 µL of Amplex Red (0.012 mM), 20 µL of SOX (0.04 U) and 20 µL of HRP (0.3 U). The absorbance (OD570) was immediately measured at 37 °C every 5 min for 30 min. All assays were performed in triplicate. Background absorbance was corrected by subtracting the value of the no-sarcosine control from all sample readings. The optimum pH range was determined in the presence of 50 µM sarcosine using the following buffer: 50 mM citrate for pH 3-5; 50 mM sodium phosphate for pH 6-7.5 and 50 mM Tris-HCl for pH 8-10. All reactions were incubated at 37 °C for 20 min. The optimal incubation time was studied by measuring the reaction every 10 min for 90 min and the reaction was performed in 50 mM sodium phosphate buffer, pH 7.5 in the presence of 100 µM sarcosine.

### Preparation of a sarcosine calibration curve

Pooled urine, collected from healthy volunteers (n = 10) who were not medicated and did not take any vitamin supplements, was used for construction of calibration curve. Each sample was screened by urinalysis. Sarcosine standard solution was prepared at a concentration of 500 µM in 50 mM sodium phosphate buffer, pH 7.5 then spiked to pooled urine at concentrations ranging from 1-200 µM (1, 2, 3, 4, 5, 10, 20, 30, 40, 50, 100 and 200 µM). The reactions were done as mentioned earlier at 37 °C for 20 min but spiked urine was used instead of sarcosine solution. All assays were performed in triplicate.

### Interference study

The potential interferences of the peroxidase-based method were evaluated by determination of sarcosine in the presence of common interference materials reported in the literature, such as ascorbic acid, glucose, uric acid and bilirubin (Kinoshita and Hiraga, 1980[20]; Fossati et al., 1983[14]; Artiss et al., 1984[2]; Kayamori et al., 2000[18]). 50 µM of sarcosine was prepared in 50 mM sodium phosphate buffer and the interference was spiked separately to correspond to a final concentration of substance (Fossati et al., 1983[14]; Yao and Zhang, 2016[38]) as shown in Table 1(Tab. 1). Moreover, significant interference of sarcosine detection could appear in the presence of other amino acids, such as glycine and alanine (Burton et al., 2013[7]). To investigate the specificity of the method, glycine or alanine was spiked individually into pooled urine to a final concentration of 50 µM. Pooled urine containing 50 µM of sarcosine was used as a control. Then sarcosine concentration was measured from the prepared spiked samples. The assays were performed in triplicate according to analytical procedure.

### Method evaluation

All experiments were performed using pooled urine. The bioanalytical method was evaluated in terms of linearity, sensitivity, limit of detection (LOD), limit of quantitation (LOQ), precision and recovery. The linearity of calibration curve was determined by linear regression analysis. A calibration curve with a correlation coefficient (R^2^) ≥0.990 was considered to be linear. Precision was examined through an assay of pooled urine spiked with sarcosine at concentrations of 1, 5, 25, and 50 μM with repeatability (n = 20 for each) and intermediate precision was determined using the spiked urine with the same concentrations of sarcosine (n = 20 for each) for 5 consecutive days. Recovery was evaluated by spiking known amounts of sarcosine (1, 5, 25 and 50 μM) into pooled urine samples. Then sarcosine concentration was measured from the prepared spiked samples in triplicate.

### Quantitation of sarcosine from urine samples

The spot urine samples from 20 PCa patients (age range 55-84 years; mean 69 years) and from 20 healthy male volunteers (age range 31-54 years; mean 39 years) were provided by the Division of Urology, Department of Surgery, Faculty of Medicine Ramathibodi Hospital, Mahidol University. Diagnosis of PCa was made by histopathological analysis after prostate biopsy subsequently. PCa patients were identified with positive biopsy results. The urine samples were centrifuged at 4 °C, 3,000 rpm for 10 min to remove the particulates. The supernatant was collected and stored at -20 °C until used for assay. 20 µL of supernatant was taken into the well plate. The reaction was performed as that of the sarcosine spiked urine samples.

### Statistical analysis

The results were expressed as mean ± SD. All statistical analyses including regression analysis, one-way ANOVA and Paired t-test were performed using GraphPad prism 6 (GraphPad Inc., San Diego, CA, USA). Statistical significance was defined as *p*-value <0.01 or <0.05.

### Ethical approval of studies and informed consent

The study protocols and the procedures for handling human samples were approved by the Committee on Human Rights Related to Research Involving Human Subjects, Faculty of Medicine Ramathibodi Hospital, Mahidol University (MURA2016/34). Written informed consents were obtained from all subjects recruited to our study.

## Results

### Optimization of two-enzyme coupled assay for sarcosine measurement

**Figure 2:**
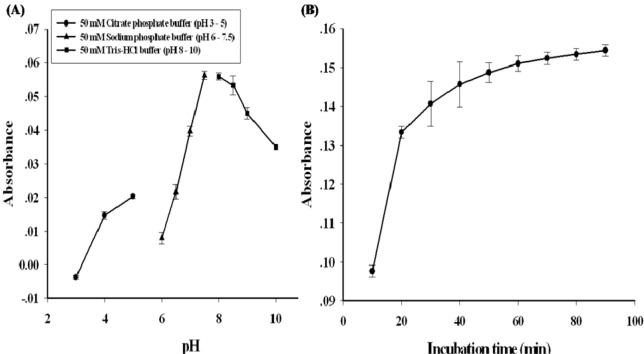
Optimization of sequential reaction of SOX and HRP. The sarcosine concentration was 50 µM. The absorbance was measured at 37 °C (A) pH effect on the reaction. (●) pH 3-5, 50 mM citrate buffer; (▲) pH 6-8, 50 mM sodium phosphate buffer; (∎) pH 8.5-10, 50 mM Tris-HCl buffer (B) Reaction time course of the assay. Each point represents the mean of three independent measurements. Error bars represent standard deviation of three independent experiments.

Since the reaction pH and time are the most important parameters of the assay, the experiments were conducted to optimize these conditions. The assay with 50 µM sarcosine standard solution was performed at various pH ranging from 3 to 10 for 20 min. The coupled assay had an optimal pH at 7.5 as shown in Figure 2A(Fig. 2). In addition, the optimal time of this assay was also investigated. 100 µM of sarcosine standard was used and the reaction was measured at every 10 min. The result showed that the steady signal was achieved in 20 min when the absorbance was constant (Figure 2B(Fig. 2)). Another important factor in method optimization is the concentrations of used enzymes. The optimized SOX and HRP concentrations were 0.04 and 0.3 U/reaction, respectively (data not shown). Accordingly, these conditions were used in the following experiments.

### Sarcosine calibration curve

**Figure 3:**
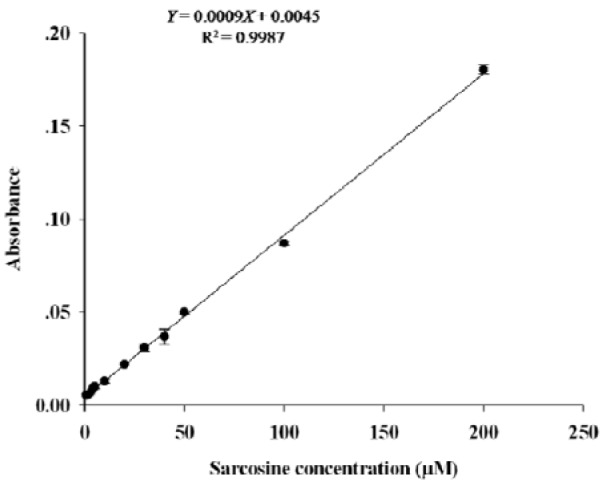
Calibration curve for sarcosine measurement in pooled urine. Absorbance was measured at 37 °C for 20 min. Experiments for each concentration were performed in triplicate.

Under optimal conditions, construction of the linear calibration curve was performed using pooled urine spiked with sarcosine in the concentration ranging from 0 to 200 µM. As shown in Figure 3(Fig. 3), the linearity of graph was obtained when sarcosine concentration was within the range of 1-200 µM and the regression equation was Y = 0.0009X + 0.0045. The correlation of coefficient (R^2^) was 0.999.

### Interference study

The interference test was performed by spiking the interested compounds into the solution containing 50 µM of sarcosine, which was then measured for sarcosine concentration. The results of this study are summarized in Table 1(Tab. 1). The colorimetric assay showed no significant interference from glucose, ascor-bic acid and bilirubin (*p*-value <0.01). However, the interference of uric acid was significantly observed.

**Figure 4:**
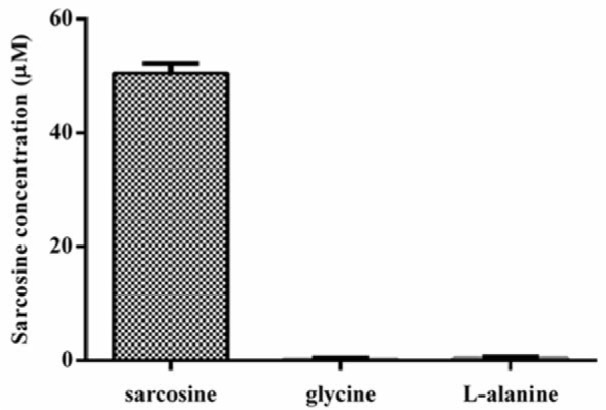
Specificity of the SOX-HRP coupled assay for sarcosine as compared to alanine and glycine. Each amino acid was spiked separately in pooled urine at final concentration of 50 µM.

According to the limitations of some methods for differentiation of sarcosine from glycine and alanine, the specificity of the developed method was evaluated. As shown in Figure 4(Fig. 4), when specified amount of glycine or alanine (50 µM) was tested, the absorbance signal could not be detected suggesting no reactions occurred. Nevertheless, with 50 µM of sarcosine, the signal increased corresponding to the amount of the tested substance. These results indicated that our assay could effectively distinguish sarcosine from other substances presented in urine without complicated sample preparation.

### Method evaluation

The linearity of this method showed a good degree of correlation between the sarcosine concentration and the absorbance. Analysis of standard curve demonstrated a nearly linear curve which confirmed the linearity of the method. The LOD and LOQ of this assay were 0.7 µM and 1 µM, respectively. Analytical precision was defined by analysis of within-run and between-run replication assays. As shown in Table 2,(Tab. 2) the coefficient of variation (%CV) at each concentration ranged from 3.52 to 18.65 % for within-run precision and from 4.45 to 18.91 % for between-run precision. These results indicated that the developed method possessed good precision since the %CV was lower than the acceptance criteria (The %CV of precision study should not exceed 15 % except the %CV at the LOQ, where it should not exceed 20 %) (Tiwari and Tiwari, 2010[32]). To assess a proportional systemic error of our method, the known amounts of sarcosine (1, 5, 25 and 50 µM) were spiked into the pooled urine. The analytical recoveries are shown in Table 3(Tab. 3). The results showed a recovery in range of 73.33-106.67 %, suggesting that sarcosine was acceptably recovered in urine. Altogether, these data indicated that our method is applicable and can be used for sarcosine detection in real urine samples.

### Quantitation of sarcosine from urine samples

**Figure 5:**
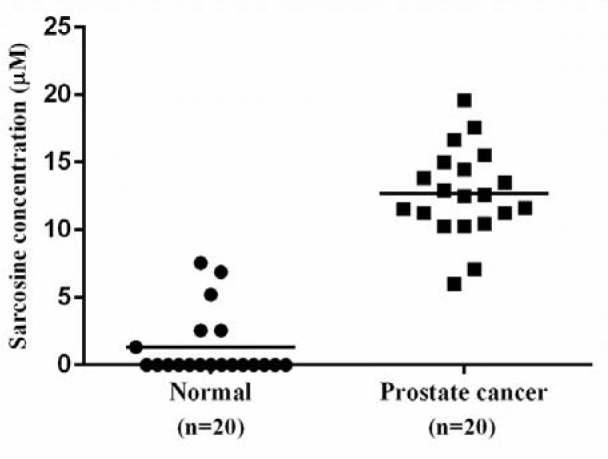
Scatter dot-plot of sarcosine concentration measuring in urine using SOX-HRP coupled assay. Horizontal solid lines are the mean values for each group of healthy subjects and PCa patients. Statistical significance was evaluated by Paired t-test (*p*-value <0.05). The mean of urinary sarcosine levels in PCa patients was significantly different from healthy donors.

The feasibility of the method for the detection of sarcosine was investigated by analyzing sarcosine in real urine samples from 20 patients with diagnosed PCa (by histological confirmation) and 20 healthy donors. The concentrations of sarcosine in the samples were calculated using the constructed calibration curve. The PCa patient characteristics and sarcosine level in urine are shown in Table 4(Tab. 4). As illustrated in Figure 5(Fig. 5), the average sarcosine concentration in urine of PCa patients (12.70 ± 3.29 µM) was statistically higher than the average concentration found in healthy subjects (1.43 ± 1.31 µM) with *p-*value <0.05. This indicates that it is possible to apply the proposed method for sarcosine detection in urine of PCa patients.

## Discussion

Sarcosine, chemically defined as the methyl derivative of glycine, formed in the mammalian body as an intermediate product. Recently, sarcosine was highlighted as one of the prostate cancer biomarker candidates that can be detected in urine (Sreekumar et al., 2009[31]). However, the opinions on sarcosine application are still controversial and publications refuting its applicability as a tumor marker have appeared (Schalken, 2010[28]; Cao et al., 2011[8]). Given that an effective diagnosis tool for sarcosine detection directly in urine is highly attractive to verify the hypothesis of marker, herein, a coupled enzyme-based sarcosine detection assay using SOX and HRP was established. In comparison with the quantitative detection of sarcosine using chromatography methods, our assay is convenient, rapid and highly specific. Moreover, compared to several electrochemical detection systems, this method is simple and applicable since it can be directly used to determine the sarcosine level from real urine sample. One of the advantages of our method is that it does not require sample pre-treatment. The LOD of our assay is estimated to 0.7 µM which is comparable to other colorimetric assays in the range of 0.5-1 µM (Perez Galende et al., 2012[25]; Xue et al., 2016[36]). Nevertheless, it is higher than the LOD of the chromatography and electrochemical-based methods. In the literature, the LOD of sarcosine has been reported at nanomolar levels; 1 and 4 nM using GC-MS and LC-MS, respectively (Jiang et al., 2010[17]; Cavaliere et al., 2011[9]), 20 nM by fluorometric method (Burton et al., 2012[6]), 45 nM by reflectometric nanosensor (Diltemiz and Uslu, 2015[13]), 30 nM using amperometric biosensor (Xue et al., 2017[35]) and 16 nM using carbon screen-print electrode electrochemical biosensor (Rebelo et al., 2014[26]). However, the sensitivity of our method is sufficient because it encompasses the concentrations of sarcosine in urine samples (1-20 µM) (Valenti et al., 2015[33]). Notably, the reaction time of our assay is approximately 20 min which is shorter than that of other methods (Cernei et al., 2012[10]; Lan et al., 2014[21]; Xue et al., 2017[35]). Moreover, the amount of enzyme used in the reaction is much less than the amount from previous reports (Perez Galende et al., 2012[25]). The optimal pH of the sequential reaction was 7.5. This result is in good agreement with previous studies that demonstrated the optimal pH of HRP and SOX to be 7 and 8.5, respectively (Chance and Maehly, 1955[11]; Guo et al., 2006[15]). Precision study exhibited the high reproducibility and repeatability of the assay suggesting that our method is potentially suitable for sarcosine determination in urine under given concentration range. Notably, the % recovery of our method was nearly 100 % indicating that the test was accurate and reliable. However, at low concentrations of sarcosine, low % recovery (73.33 %) has been observed. This result suggested that the assay should be performed thoroughly when the small amount (≤1 µM) of urinary sarcosine was present. It is well known that some substances in urine such as glucose, ascorbic acid, bilirubin, as well as uric acid often affect enzymatic methods that utilize peroxidase. In this case, ascorbic acid, glucose and bilirubin at studied concentrations did not significantly interfere to our system. Nevertheless, uric acid at concentrations in range of 0.2-4.4 mM was found to influence the measurement. Therefore, in order to obtain high accuracy of sarcosine determination, urine samples containing high concentration of uric acid should be carefully tested. Elimination of uric acid prior to sarcosine measurement might be necessary. In the literature, the separation and determination of sarcosine in biological matrix is crucial due to its similarity with high abundant alanine isomer, identical molecular mass (Martinez-Lozano and Rus, 2012[23]). However, this study demonstrated that the SOX-HRP coupled assay has an excellent selectivity for sarcosine compared to other amino acids, alanine and glycine. This could be due to the active site of SOX enzyme that is highly specific for its substrate. In order to evaluate the efficiency of the method, urine samples from healthy subjects and PCa patients were measured for sarcosine concentration. The means of sarcosine concentrations in PCa patients and normal subjects were statistically different. The mean of sarcosine concentrations in PCa patients was approximately 9 times greater than that of normal subjects. The urinary sarcosine level is in the same range with the previous reports (Shamsipur et al., 2013[29]; Lan et al., 2014[21]), suggesting that the proposed assay is effective and applicable for sarcosine determination in urine.

## Conclusion

In this study, a sensitive and specific SOX-HRP coupled assay for urinary sarcosine determination was successfully developed. Under optimized conditions, the assay exhibited good accuracy (%recovery) and precision (%CV). The advantages of our assay include: (a) there is no requirement for sample preparation or dilution which the samples could be applied directly to the reaction; (b) the assay is a method in which large numbers of samples can be tested simultaneously in 96-well microtiter plate; (c) the assay is a colorimetric-based method monitoring by spectrophotometer which is generally available at most laboratories making the test more appropriate for routine application; (d) the reaction time is short, the results can be obtained in only 20 min; (e) the interferences, such as ascorbic acid, glucose and bilirubin did not significantly affect the assay. According to these advantages, this assay is a potential method that could be used to quantify the concentration of sarcosine in urine. Importantly, the method is promising to discriminate PCa patients from healthy subjects based on different concentration of sarcosine in urine. However, further experiments with more sample size must be performed to evaluate whether sarcosine is effective as a urinary biomarker for PCa. The present study not only gained an insight into the determination for sarcosine concentration in urine but also opened up an opportunity for accurate diagnosis of prostate cancer.

## Conflict of interest

The authors declare that they have no conflict of interest.

## Acknowledgements

This work was supported by National Research Council of Thailand and Health Systems Research Institute [grant number HSRI 60-026].

## Figures and Tables

**Table 1:**
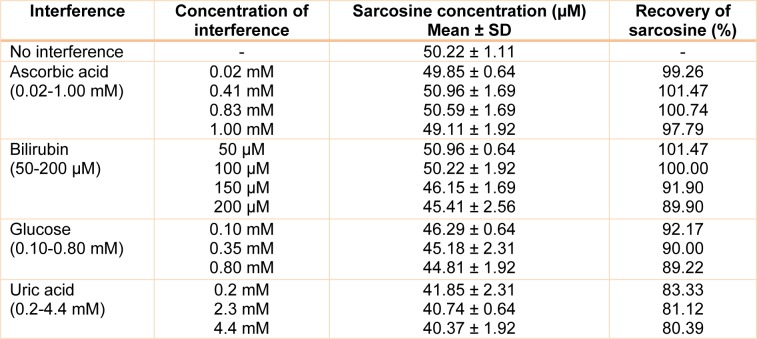
Interference of some compounds on the determination of 50 µM sarcosine (*p*-value <0.01)

**Table 2:**
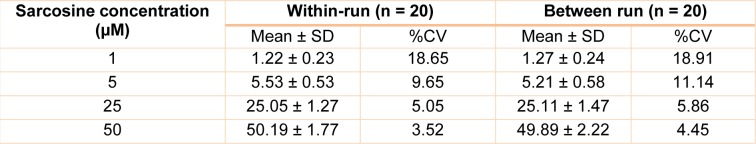
The within-run and between-run precision of the coupled enzymatic assay for sarcosine measurement

**Table 3:**
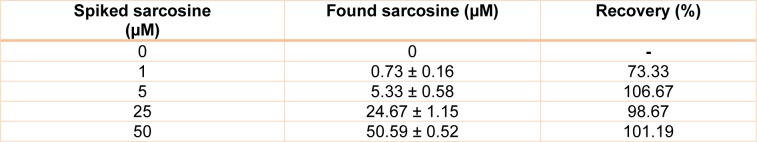
Spiked recovery of sarcosine in pooled urine

**Table 4:**
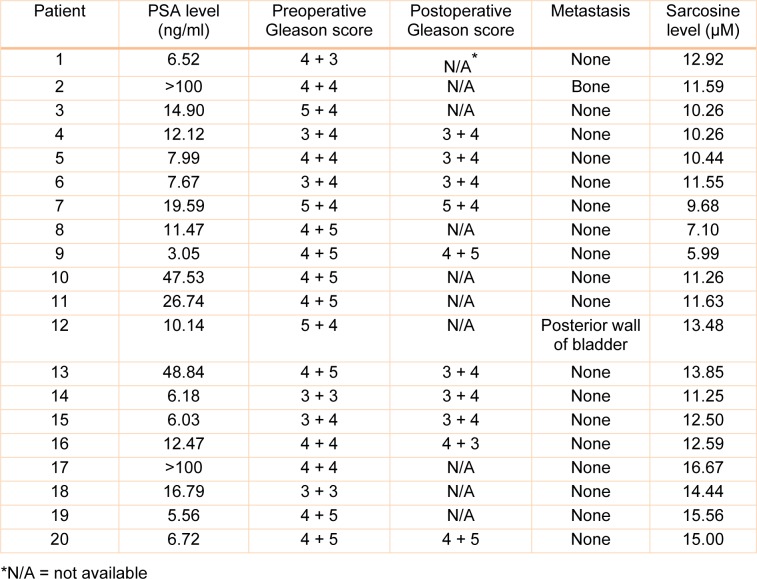
PCa patient characteristics and sarcosine level in urine

## References

[1] Ankerst DP, Thompson IM (2006). Sensitivity and specificity of prostate-specific antigen for prostate cancer detection with high rates of biopsy verification. Arch Ital Urol Androl.

[2] Artiss JD, McEnroe RJ, Zak B (1984). Bilirubin interference in a peroxidase-coupled procedure for creatinine eliminated by bilirubin oxidase. Clin Chem.

[3] Aslan G, Irer B, Cimen S, Goktay Y, Celebi I, Tuna B (2011). The performance of abnormal digital rectal examination for the detection of prostate cancer at stratified prostate specific antigen levels. Open J Urol.

[4] Bianchi F, Dugheri S, Musci M, Bonacchi A, Salvadori E, Arcangeli G (2011). Fully automated solid-phase microextraction-fast gas chromatography-mass spectrometry method using a new ionic liquid column for high-throughput analysis of sarcosine and N-ethylglycine in human urine and urinary sediments. Anal Chim Acta.

[5] Biavardi E, Tudisco C, Maffei F, Motta A, Massera C, Condorelli GG (2012). Exclusive recognition of sarcosine in water and urine by a cavitand-functionalized silicon surface. Proc Natl Acad Sci U S A.

[6] Burton C, Gamagedara S, Ma Y (2012). A novel enzymatic technique for determination of sarcosine in urine samples. Anal Methods.

[7] Burton C, Gamagedara S, Ma Y (2013). Partial enzymatic elimination and quantification of sarcosine from alanine using liquid chromatography-tandem massspectrometry. Anal Bioanal Chem.

[8] Cao DL, Ye DW, Zhu Y, Zhang HL, Wang YX, Yao XD (2011). Efforts to resolve the contradictions in early diagnosis of prostate cancer: comparison of different algorithms of sarcosine in urine. Prostate Cancer Prostatic Dis.

[9] Cavaliere B, Macchione B, Monteleone M, Naccarato A, Sindona G, Tagarelli A (2011). Sarcosine as a marker in prostate cancer progression: a rapid and simple method for its quantification in human urine by solid-phase microextraction-gas chromatography–triple quadrupole mass spectrometry. Anal Bioanal Chem.

[10] Cernei N, Zitka O, Ryvolova M, Adam V, Masarik M, Hubalek J (2012). Spectrometric and electrochemical analysis of sarcosine as a potential prostate carcinoma marker. Int J Electrochem Sci.

[11] Chance B, Maehly AC (1955). Assay of catalases and peroxidases. Methods Enzymol.

[12] Clint Cary K, Cooperberg MR (2013). Biomarker in prostate cancer surveillance and screening: past, present, and future. Ther Adv Urol.

[13] Diltemiz SE, Uslu O (2015). A reflectometric interferometric nanosensor for sarcosine. Biotechnol Prog.

[14] Fossati P, Prencipe L, Berti G (1983). Enzymic creatinine assay: a new colorimetric method based on hydrogen peroxide measurement. Clin Chem.

[15] Guo K, Ma X, Sun G, Zhao Y, Li X, Zhao W (2006). Expression and characterization of a thermostable sarcosine oxidase (SOX) from Bacillus sp. in Escherichia coli. Appl Microbiol Biotechnol.

[16] Jamaspishvili T, Kral M, Khomeriki I, Student V, Kola Z, Bouchal J (2010). Urine marker in monitoring for prostate cancer. Prostate Cancer Prostatic Dis.

[17] Jiang YQ, Cheng XL, Wang CA, Ma YF (2010). Quantitative determination of sarcosine and related compounds in urinary samples. Anal Chem.

[18] Kayamori Y, Katayama Y, Urata T (2000). Nonenzymatic elimination of ascorbic acid in clinical samples. Clin Biochem.

[19] Khan AP, Rajendiran TM, Ateeq B, Asangani IA, Athanikar JN, Yocum AK (2013). The role of sarcosine metabolism in prostate cancer progression. Neoplasia.

[20] Kinoshita T, Hiraga Y (1980). A fluorophotometric determination of serum creatinine and creatine using a creatinineamidohydrolase-creatineamidinohydrolase-sarcosine oxidase-peroxidase system and diacetyldichlorofluorescin. Chem Pharm Bull (Tokyo).

[21] Lan J, Xu W, Wan Q, Zhang X, Lin J, Chen J (2014). Colorimetric determination of sarcosine in urine samples of prostatic carcinoma by mimic enzyme pallidum nanoparticles. Anal Chim Acta.

[22] Loeb S, Catalona WJ (2010). Prostate cancer: utility of the risk indicator model in screening. Nat Rev Urol.

[23] Martinez-Lozano P, Rus J (2012). Separation of isomers L-alanine and sarcosine in urine by electrospray ionization and tandem differential mobility analysis-mass spectrometry. J Am Soc Mass Spectrom.

[24] Meyer T, Fox S, Issaq H, Xu X, Chu L, Veenstra T (2011). A reproducible and high-throughput HPLC/MS method to separate sarcosine from α- and β-alanine and to quantuify sarcosine in human serum and urine. Anal Chem.

[25] Perez Galende P, Manzano Munoz T, Roig MG, Garcia de Maria C (2012). Use of crude extarct of lentil plant (Lens culinaris Medikus) in peroxidase-based analyses: fast kinetic determination of hydrogen peroxide and sarcosine in urine. Anal Bioanal Chem.

[26] Rebelo TS, Pereira CM, Sales MG, Noronha JP, Costa-Rodrigues J, Silva F (2014). Sarcosine oxidase composite screen-printed electrode for sarcosine determination in biological samples. Anal Chim Acta.

[27] Roobol MJ, Zappa M, Maatanen L, Ciatto S (2007). The value of different screening tests in predicting prostate biopsy outcome in screening for prostate cancer data from a multicenter study (ERSPC). Prostate.

[28] Schalken JA (2010). Is urinary sarcosine useful to identify patients with significant prostate cancer? The trials and tribulations of biomarker development. Eur Urol.

[29] Shamsipur M, Naseri MT, Brabi M (2013). Quantification of candidate prostate cancer metabolite biomarkers in urine using dispersive derivatization liquid-liquid microextraction followed by gas and liquid chromatography-mass spectrometry. J Pharm Biomed Anal.

[30] Smith RA, Cokkinides V, Eyre HJ, American Cancer Society (2004). American Cancer Society guidelines for the early of cancer. CA Cancer J Clin.

[31] Sreekumar A, Poisson LM, Rajendiran TM, Khan AP, Cao Q, Yu JD (2009). Metabolomic profiles delineate potential role for sarcosine in prostate cancer progression. Nature.

[32] Tiwari G, Tiwari R (2010). Bioanalytical method validation: An updated review. Pharm Methods.

[33] Valenti G, Rampazzo E, Biavardi E, Vallani E, Fracasso G, Marcaccio M (2015). An electrochemiluminescence-supramolecular approach to sarcosine detection for early diagnosis of prostate cancer. Faraday Discuss.

[34] Votyakova TV, Reynolds IJ (2004). Detection of hydrogen peroxide with Amplex Red: interference by NADH and reduced glutathione auto-oxidation. Arch Biochem Biophys.

[35] Xue Z, Wang H, Rao H, He N, Wang X, Liu X (2017). Amperometric indicator displacement assay for biomarker monitoring: Indirectly sensing strategy for electrochemically inactive sarcosine. Talanta.

[36] Xue Z, Yin B, Wang H, Li M, Rao H, Liu X (2016). An organic indicator functionalized graphene oxide nanocomposite-based colorimetric assay for the detection of sarcosine. Nanoscale.

[37] Yadav S, Devi R, Bhar P, Singhla S, Pundir CS (2012). Immobilization of creatininase, creatinase and sarcosine oxidase on iron oxide nanoparticles/chitosan-g-polyaniline modified Pt electrode for detection of creatinine. Enzyme Microb Technol.

[38] Yao Y, Zhang C (2016). A novel screen-printed microfluidic paper-based electrochemical device for detection of glucose and uric acid in urine. Biomed Microdevices.

[39] Zhou M, Diwu Z, Panchuk-Voloshina N, Haugland RP (1997). A stable nonfluorescent derivative of resorufin for the fluorometric determination of trace hydrogen peroxide: applications in detecting the activity of phagocyte NADPH oxidase and other oxidases. Anal Biochem.

